# The Nucleic Acid Database: Present and Future

**DOI:** 10.6028/jres.101.026

**Published:** 1996

**Authors:** Helen M. Berman, Anke Gelbin, Lester Clowney, Shu-Hsin Hsieh, Christine Zardecki, John Westbrook

**Affiliations:** Department of Chemistry, Rutgers University, Piscataway, NJ 08855-0939

**Keywords:** database management, Macro-molecular Crystallographic Information File, nucleic acid structure

## Abstract

The Nucleic Acid Database is a relational database containing information about three-dimensional nucleic acid structures. The methods used for data processing, structure validation, database management and information retrieval, as well as the various services available via the World Wide Web, are described. Plans for the future include greater reliance on the Macromolecular Crystallographic Information File for both data processing and data management.

## 1. Introduction

The Nucleic Acid Database (NDB) [1] was established in 1991 as a resource for specialists in the field of nucleic acid structure. Its purpose was to gather all the structural information about oligonucleotides that had been obtained from x-ray crystallographic experiments and to organize them in such a way that it would be easy to retrieve the coordinates, the information about the experimental conditions used to derive these coordinates, and the structural information that could be derived from these coordinates. It was clear from the beginning that many of the users of these data would not themselves be crystallographers, and that the information provided by the database had to be presented in such a way as to maximize its utility for various types of modeling and structure prediction.

As the project progressed, many new technologies developed that presented challenges and opportunities. These include the development of the standard interchange format for handling crystallographic data, called the Macromolecular Crystallographic Information File (mmCIF), and the explosive use of the World Wide Web (WWW).

## 2. Database Contents

Structures available in the NDB include RNA and DNA oligonucleotides with two or more bases. These oligonucleotides may be complexed with drugs and ions. Structures of larger nucleic acid containing crystals, including protein-DNA and protein-RNA structures, are also curated and included in the archive. Table 1 shows the current holdings of the NDB.

Current literature is scanned on a regular basis, and structures suitable for inclusion in the NDB are noted. Coordinates sets are retrieved from the Protein Data Bank (PDB) [2] and are then filtered into the NDB format. In the case of oligonucleotides not complexed with proteins, coordinate sets submitted by the author for submission into the PDB are processed. Starting in January 1996, the NDB became a direct deposition site for these oligonucleotide structures.

In addition to coordinate data, information relevant to the crystallographic experiment is abstracted from the primary literature for inclusion into the database. These include crystallization conditions, refinement statistics and data collection statistics. Other derived information, such as the distances, angles, torsion angles, and base morphology parameters, is calculated from the coordinate data and placed in the database. Tables 2a and 2b list summaries of the information currently in the NDB.

## 3. Data Processing

### 3.1 Data Entry and Integrity Checks

The scheme for data processing is given in Figs. 1a and 1b. A set of filter programs have been developed that allow this process of data entry and integrity checking to be highly automated. A key feature of the system is the use of a template based on mmCIF. A template is a C1F data file that includes definition and example information from the mmCIF dictionary which serve as comments preceding each data category. The CIF template is a skeleton file that is easily used with a text editor. The NDB has created software tools to populate the CIF template with data from a variety of file formats. Items which cannot be loaded electronically are flagged for later manual entry. This method allows the NDB to work with a large variety of formats. For example, all items that are fully parseable from the PDB can be loaded into a template. The rest of the information, provided in the manuscripts or in the text parts of the PDB file, can be entered by a data curator. Files in completely different formats can be handled by reordering the mmCIF tokens. After the template is completed, new items that can be derived from the coordinate sets, such as the DNA sequence, are added using the NDB-filter programs. Checks are built into the filter programs that ensure that the coordinates have standard ordering, and that the nomenclatures of both the polymers and the ligands are consistent. In addition, programs have been written that allow many of the data items to be automatically extracted from the commonly used refinement programs for nucleic acids. The use of these filter programs permits the data processing procedure, including checking, to be completed in about 3 h per structure. The rate limiting step is the gathering of missing data items that are not included in any of the standard computer files used as input into data processing.

The result of these processes is a flat file in the NDB format which is ready to be loaded into the database.

### 3.2 Database Management

Once the first level of checks have been made on the data, they are entered into a relational database using SYBASE as the database management system. Over 60 tables are created in the original raw data. A simple menu driven program, NDBquery allows the user to interact with the database using a natural language rather than SQL. The same program manages the calculation of derived quantities, including distances, angles, torsion angles and base morphology parameters, for each structure that are then loaded into the database.

## 4. Information Retrieval

### 4.1 Constraint Generation

The NDB uses a two phase system to query the database. In the first pass, the structural features that are to be considered are selected. Any of the data items stored in the database can serve as a selection constraint. For example, it is possible to select structures of a particular type which have torsion angles in a particular range and which have been determined by a particular author. Two examples of the use of structure selection constraints are presented in Tables 3a and 3b.

**Fig. 2:**
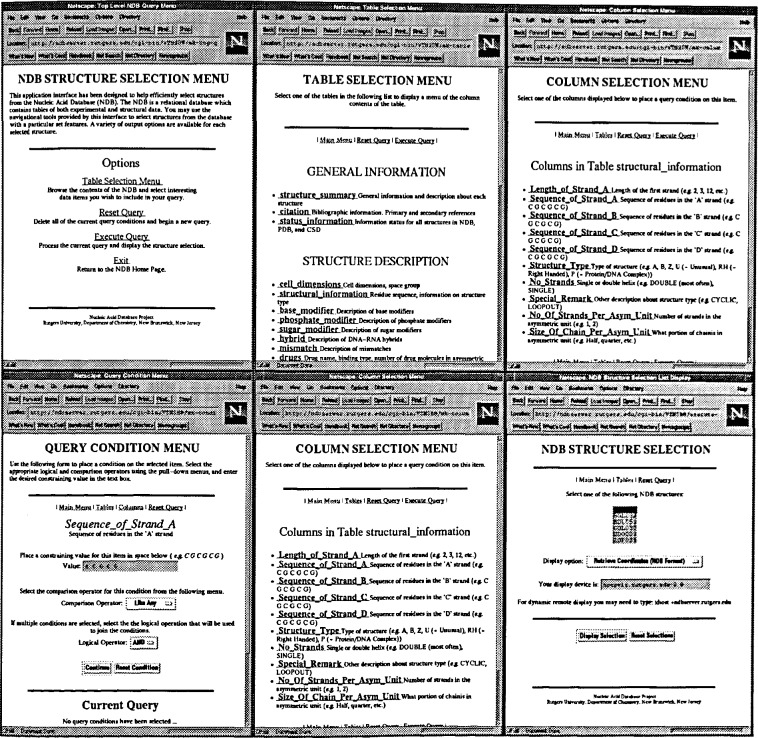
Sequence for a simple query, i.e., choosing structures that contain the specific sequence ACGCG using the WWW Interface, version 2.0 (October 1995).

It is possible to use either the menu driven interface to NDBquery or the WWW forms based system to gencrate selection constraints. The advantage of the latter method is that it places no restrictions on the user other than the ability to use the World Wide Web using either Netscape or Mosaic. A sample query using the WWW access is shown in Fig. 2.

### 4.2 Report Generation

**Fig. 3:**
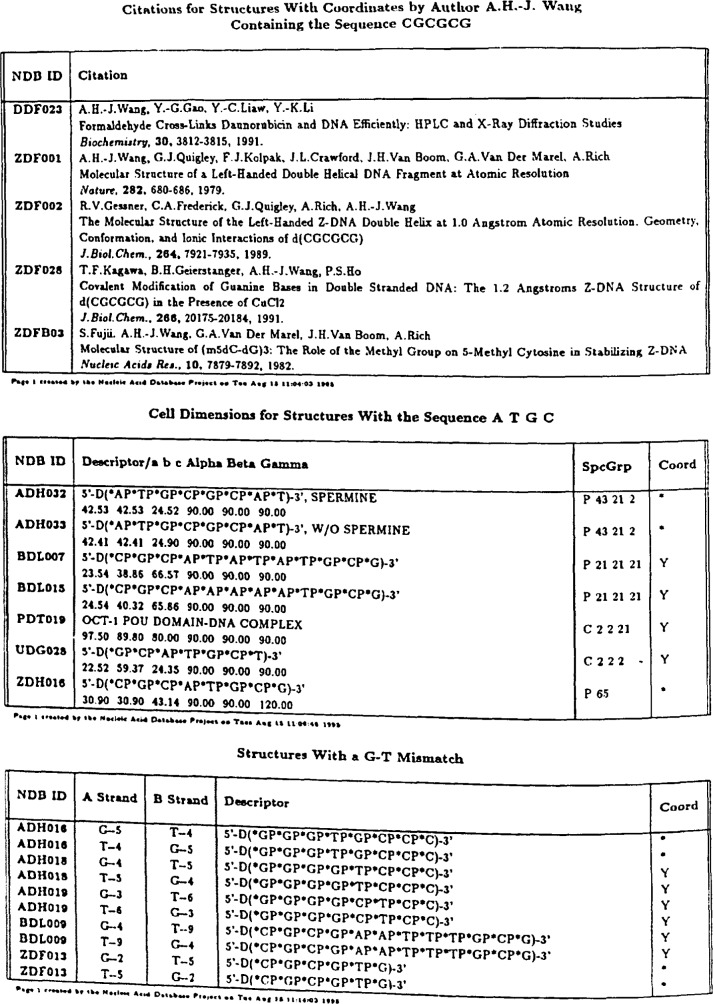
Examples of Postscript reports created by NDBquery.

Once the selection constraints are defined, a large variety of reports can be generated that describe any of the properties that are stored in the database. The simplest type of report is the list of coordinates for the selected structures. In addition, the NDBquery program produces reports in a wide variety of formats. Tabular reports such as those shown in Fig. 3 can be produced in cither ASCII or PostScript formats.

**Fig. 4:**
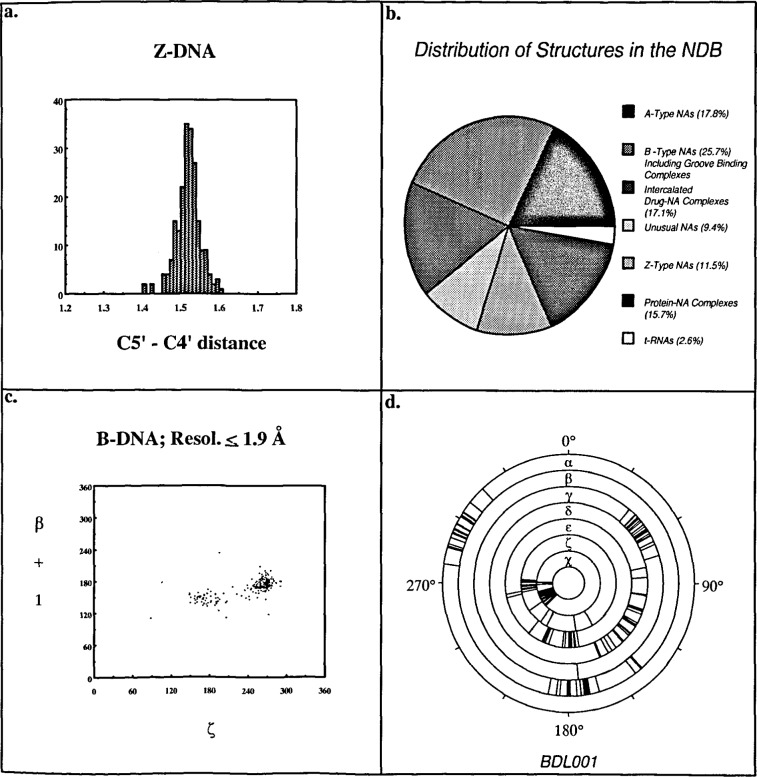
Examples of Postscript graphs created by NDBquery. (a) Histogram of the distribution of the C5′–C4′ bond lengths in Z-DNA. (b) Pie chart showing the distribution of structure types in the NDB. (c) Scatterchart of *ζ* vs *β*-torsion angles in successive residues of high resolution B-DNA structures, (d) Conformation wheel of the observed torsion angles in the Dickerson dodecamer, BDL001 [11].

Graphical reports relating any two properties can be generated. It is possible to produce scatter charts, histograms, and pic charts that can be used to analyze the properties of the structures contained within the database. These report features were used to examine the frequency distributions as well as the correlations of torsion angles of the three classes of DNA duplexes. In order to automate this type of survey, batch query capabilities were built into the system. Examples of graphical outputs are shown in Fig. 4.

**Fig. 5:**
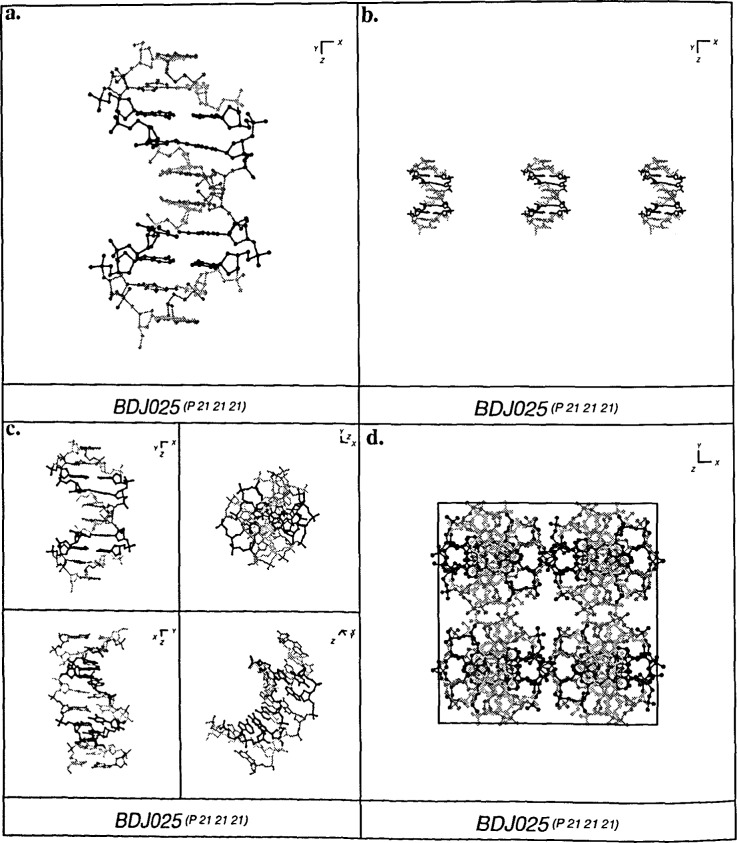
Examples of Postscript molecular graphics created by NDBquery for the self-complimentary duplex d(CGATCGATCG)_2_, BDJ025 [12]. (a) Ball and stick, (b) Stercotriptych [13J. (c) Four representative views, (d) Packing diagrams.

The NDBquery program also produces molecular graphics in a variety of formats. Structures can be depicted using color codes for the properties of the atoms or residues. Automatic packing pictures are generated in PostScript format using NDBquery and in raster form using NDBview [3]. Various types of representations, including ball and stick and Van der Waals spheres, are available (Fig. 5).

There are provisions for detailed formatting so that a complete set of publication quality reports for a set of structures can be produced. To simplify the query process, some standard and commonly used queries are saved and made available for the user. In addition, the user may save her own queries to be used repeatedly for a particular project.

The WWW forms based interface also allows for report generation. Coordinates may be retrieved in mmCIF, NDB or PDB format. It is also possible to retrieve an Atlas page (see later) and to view the structure using a dynamic viewer. The latest version of the WWW Interface can also create tabular reports based on any of the features contained in the database.

Beginning from the upper left:

- The Table Selection Menu from the NDB Structure Selection Menu is chosen.
- The Structural_information menu is selected from the Table Selection Menu.
- Sequence_of_Strand_A is selected from the Column Selection Menu.
- The desired sequence, A C G C G, is entered in capital letters with spaces separating each residue in the provided field. To move to the next step, the Continue bar, is selected.
- Once all of the desired constraints are selected, Execute Query is pressed from the top of the Column Selection Menu.
- A list of the NDB identifiers of the structures containing the sequence ACGCG is presented. The user may now:
  Retrieve coordinates in NDB Format
  Retrieve coordinátes and the bibliographic information in NDB Format (Full Entry)
  Retrieve coordinates in PDB Format
  Display the structure using a remote viewer (launching RasMol viewer on ndbserver)
  Display the structure using a local viewer (launching your own viewer)
  Display the Atlas Entry for the structure

## 5. Structure Validation

### 5.1 Standard Dictionaries

A major goal of the NDB Project is to develop and distribute methods to validate the structural features of nucleic acids. The first step in this process was to develop standard dictionaries of me valence geometry of oligonucleotides. Various dictionaries had been used by refinement programs, and it was felt that a new set of standard numbers should be derived. This was done by using very high resolution structures from the Cambridge Structural Database (CSD) [4]. Very accurate values were derived for the bases [5] and for the two standard conformations of the sugars [6]. The limited size of the small molecule sample made derivation of the phosphate geometry less satisfying. Nonetheless, it was possible to use these values and their standard deviation to develop force constants that could be used with X-PLOR [7]. Structures refined with these new values yielded much reduced rms deviations between the refined and the target geometries.

The NDB uses these standard values for the valence geometry to check structures contained within the database.

### 5.2 NDB Surveys

There are now a sufficient number of structures contained in the NDB to be able to develop expected values of various structural parameters. Surveys have been done for all of the geometric properties, including bond distances, bond angles, and torsion angles [8]. The structures contained within the database had valence geometries which, for the most part, did not deviate from the small molecule results. Indeed, subtle features related to the differences in valence geometry between the C3′ *endo* sugar pucker found in A-DNA and the C2′ *endo* sugar conformation found in B-DNA were reflected in the survey. The only features that showed some differences between what was observed in the small molecule sample and the oligonucleotides were observed in the phosphodiester geometry. These effects may be very real and it is possible that in the future these values will be used to validate the phosphodiester geometry.

**Fig. 6:**
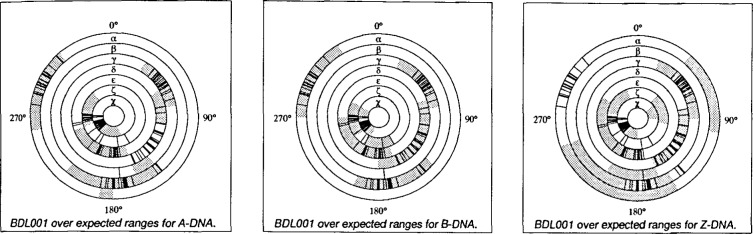
Torsion angle wheels for the B-DNA structure BDL001 [11]. The expected ranges for A-DNA, B-DNA and Z-DNA are shaded. In this example, all of the values for the torsion angles fall completely within the B-DNA range.

The torsion angle survey [8] resulted in the first experimentally derived set of ranges of torsion angles for this class of molecules. These values may be of great use in restrained refinement and in model building. The NDB has also created a "scoring system" that allows the conformation type of a DNA duplex to be assigned and checked against the assignment by the author (Fig. 6).

## 6. Distribution

### 6.1 World Wide Web

**Fig. 7:**
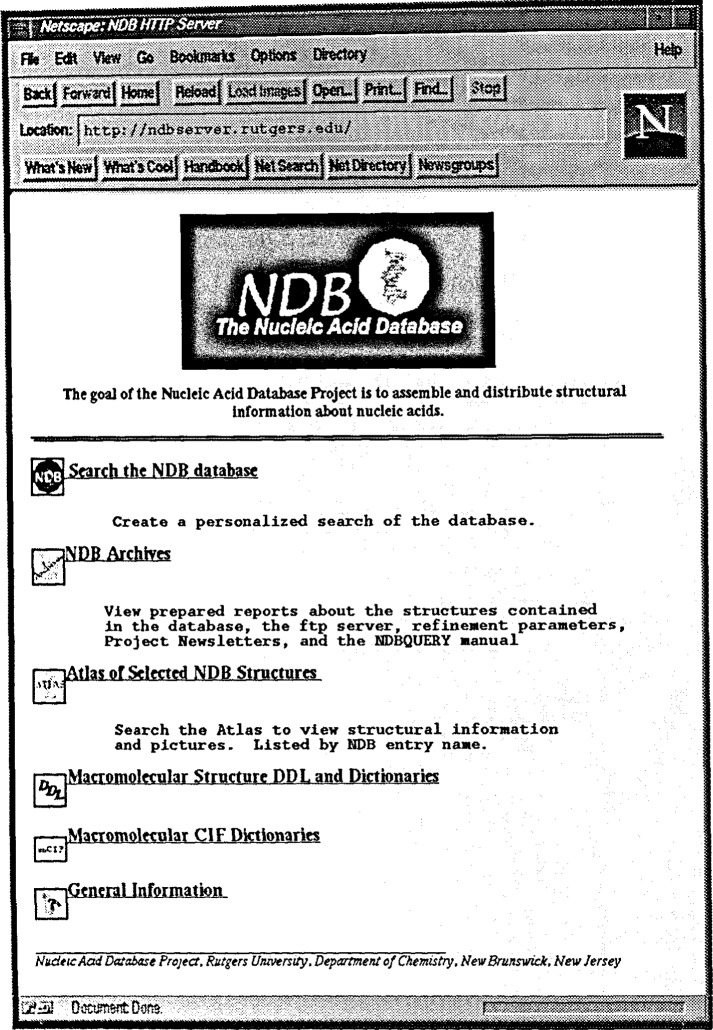
The NDB Homepage (available at http://ndbserver.rutgers.edu and is mirrored at the European Bioinformatics Institute at http://www.ebt.ac.uk/NDB/).

The NDB is available electronically via the World Wide Web (http://ndbserver.rutgers.edu and http://www.ebi.ac.uk/NDB/). In addition to providing direct query access through the forms based interface, the homepage (Fig. 7) offers access to a variety of other information.

**Fig. 8:**
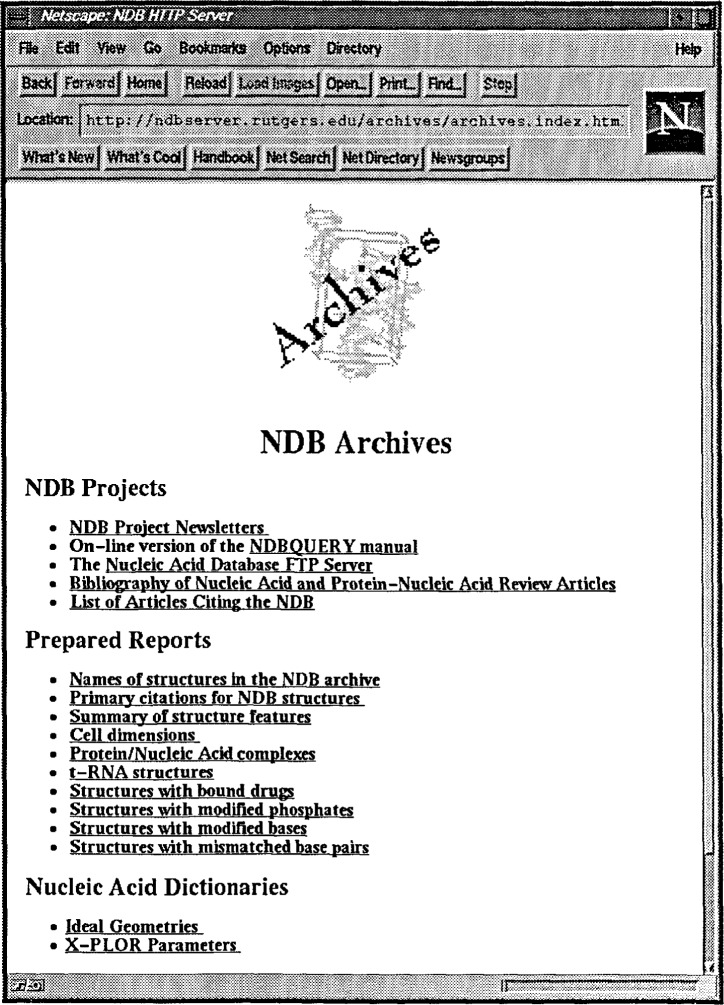
The NDB Archives Page.

The NDB Archives maintain information about the NDB Project, which include the Project Newsletters and the NDBquery manual, as well as bibliographies of review articles and research articles that cite the NDB (Fig. 8). The Archives also furnish prepared reports about the structures in the database, including citations, structural features and cell dimensions. There are tables contained in the database of information about the various subcategories of structures, including DNA, RNA, nucleic acid-protein complexes, and nucleic acid-drug complexes. Nucleic Acid Dictionaries are included in me NDB Archives, and feature X-PLOR parameters and ideal geometries for DNA/RNA bases and sugar phosphates [5–7]. The archives are updated frequently, and can be accessed via the WWW or by anonymous ftp.

**Fig. 9:**
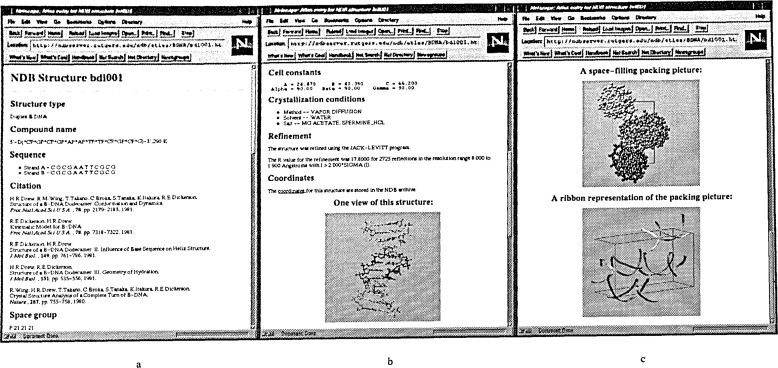
An Atlas entry for the first B-DNA crystal structure, BDL001 [13]. (a) The top of the entry page shows the structure type, compound name, sequence, citation, and space group, (b) Also included in the atlas entry are cell constants, crystallization conditions, refinement, and a link to the coordinate file for the structure. A ball and stick representation of the structure is color coded by sequence, with thymine in blue, adenine in red, cytosine in yellow, and guanine in green. (c) The space filling and ribbon representations of the unit cell are color coded in terms of the symmetry related molecules.

Another feature of the NDB WWW site is the Atlas of Selected NDB Structures (Fig. 9). Each Atlas entry highlights die bibliographical, structural and experimental information about each structure, as well as providing pictures from different views and a link to the coordinate file for the structure.

Also included on the NDB home page is die documentation for both the Dictionary Description Language for Macromolecular Structure (DDL) [9] and the Macromolecular Crystallographic Information File (mmCTF) [10] For more information on the NDB Project and other related sites, me General Information page provides a brief summary of the information in this article and useful links to other sites.

### 6.2 Newsletter

Published four times a year, the NDB Project Newsletter provides a list of recently released structures and any updates on the project itself. To subscribe, a message should be sent to ndblib@ndbserver.rutgers.edu with the subject "subscribe."

### 6.3 Custom Queries

Specialized and custom queries that are unavailable through the forms based interface on me WWW may be requested by sending mail to ndbadmin@ndbserver.rutgers.edu.

These requests can be for tabular reports containing the derived quantities available in the database, such as bond lengths, valence angles, torsion angles, or base morphology parameters. Molecular graphics, including packing pictures, may also be requested.

## 7. Future

The NDB will continue to develop and expand its scope. Most notable will be die full integration of mmCIF into all aspects of data processing. The NDB plans to provide more resource materials to researchers in die field, as well as to casual “surfers” who may want to learn more about nucleic acid structure.

### 7.1 Data Processing

**Fig. 10:**
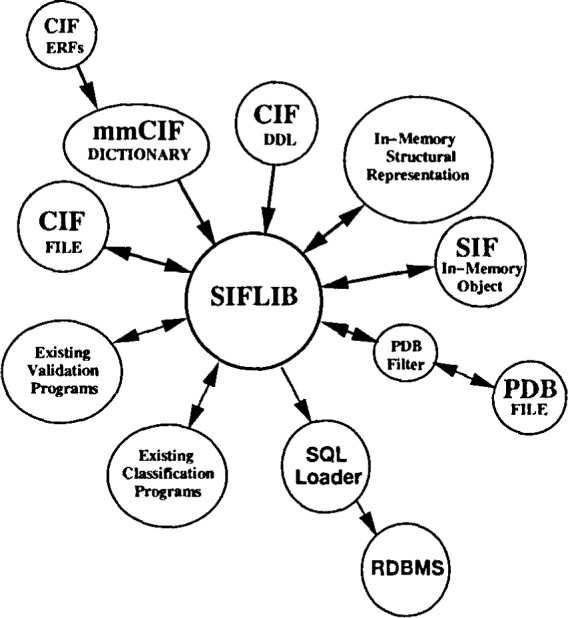
Functional diagram of SIFLIB illustrating the interaction of this utility library with a variety of other applications. The figure highlights the role of SIFLIB in encapsulating access to CIF Format data and dictionaries from calling applications.

The NDB Project has served as a test bed for the method of data description embodied in the Macromolecular Crystallographic Information File (mmCIF) and has employed mmCIF as an interchange format using a locally developed dictionary. At each stage in the evolution of the mmCTF dictionary, software tools have been developed by me NDB to evaluate the extent to which each dictionary would facilitate the automated processing of data. The result of this development is the collection of software tools called SIFLIB (Fig. 10).

SIFLIB is a class library of tools which were de֊ signed to encapsulate operations on CIF format files and dictionaries. We have chosen to name this library using me more general terminology Structure Information File (SIF) to emphasize mat these tools could be used with dictionaries for experimental techniques other man crystallography (e.g., NMR). SIFLIB was developed in conjunction with the Dictionary Description Language (DDL) Version 2.1 [9] on which the mmCIF dictionary is based. Some of me functions performed by SIFLIB include: reading and writing CIF format data files and dictionaries; reading and writing individual CIF data items; data integrity checking of CIF data items; and navigation through the CIF schema.

As the first version of the mmCIF dictionary nears completion, the NDB is converting its data processing system based on the mmCIF local dictionary to a system which is based on the data representation in the mmCIF dictionary. The core of this conversion is the integration of SIFL1B into the NDB data processing scheme as shown in Fig. 10. The key feature of this new data processing scheme is that it takes full advantage of the data description provided by the mmCIF dictionary which now contains all of the information necessary to perform detailed integrity checks for individual data items as well as for the relationships between data items.

### 7.2 Validation

As a result of the surveys of both the NDB and CSD databases, dictionaries of standard covalent geometries and observed ranges of other structural features have been compiled.

These dictionaries provide the foundation for the continued development of structural validation tools that will be used as benchmarks to evaluate each structure submitted to the NDB.

mmCIF provides a mechanism for standardizing the encoding of structural standards and other lengthy tabulations reference data in External Reference Files (ERFs). Information stored in ERFs can be accessed using the same software (SIFLIB) as other CIF data. We plan to integrate structural ERFs automatically into the NDB data processing scheme (Fig. 10).

### 7.3 Information Retrieval

The recently developed WWW interface to the NDB database provides the structure selection features of the more robust menu-driven interface, NDBquery. An enhanced version of the WWW interface that will provide both structure selection as well as report generation has recently been released.

**Fig. 11:**
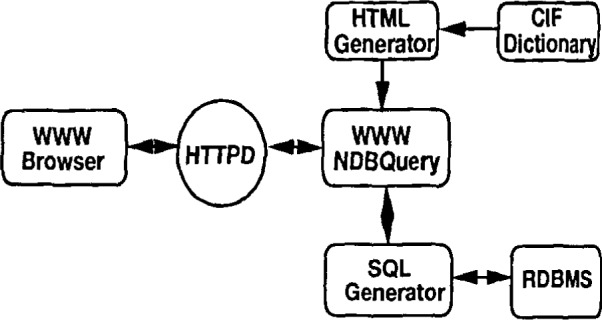
Schematic view of the NDB WWW forms based interface. The WWW version of NDBquery is called by the WWW server and provides the server with a description of the contents of the NDB database, which is presented as a *set of menu* selections. The WWW interface also manages the construction of SQL queries and all communication with the NDB database.

The WWW interface is shown schematically in Fig. 11. The figure highlights the underlying use of a CIF dictionary to describe the database schema for the WWW interface.

## Figures and Tables

**Fig 1a:**
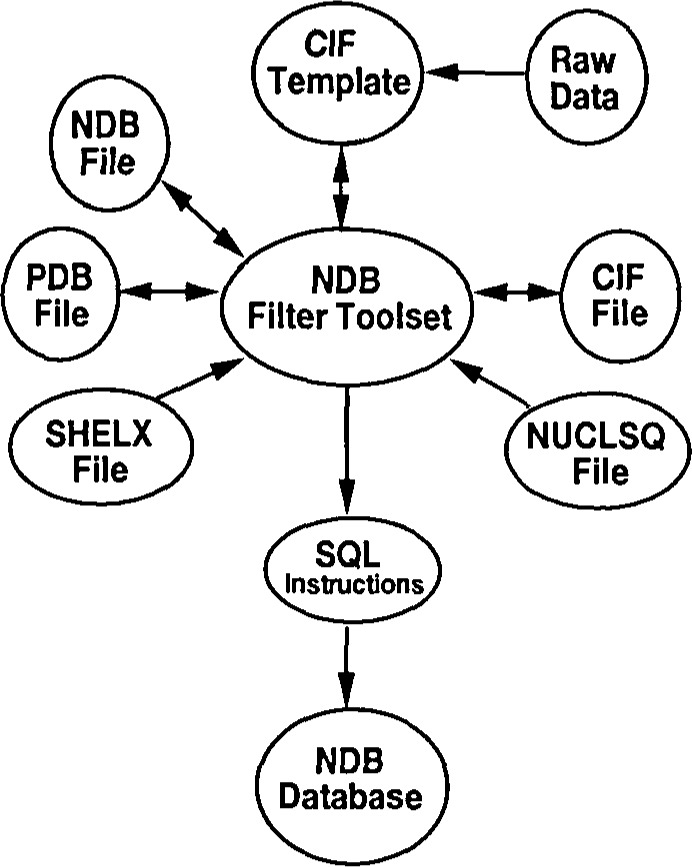
A schematic diagram of NDB data processing that illustrates the central role of the NDB filter software in automating the exchange of information between a variety of input formats and the mmCIF template and data file archival format.

**Fig 1b:**
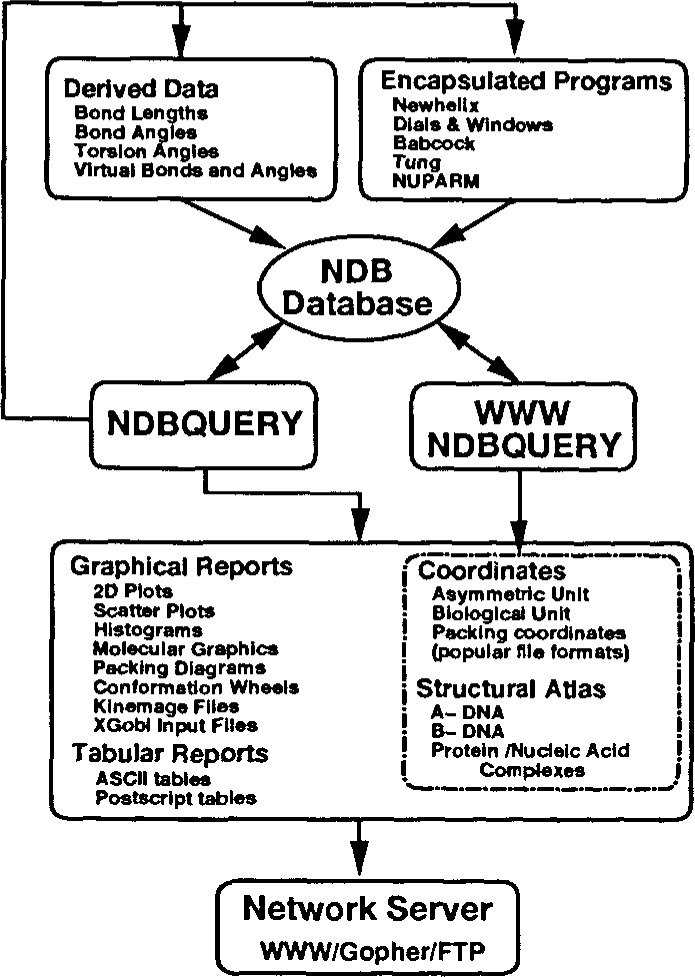
Schematic view of the data flow in and out of the NDB database as of October 1995. The figure illustrates the generation of derived structural features by the NDBquery program using both internal functions and encapsulated external programs. The collection of report types created by NDBquery is also shown. All of these reports are accessible via the NDB network server.

**Table 1:** NDB holdings as of October 1995 408 structures (390 released)

Structure Type	Number
A-DNA	51
DNA/RNA Hybrid	11
A-RNA	10
DNA-Drug Complexes	93
B-DNA	66
RNA-Drug Complexes	19
Z-DNA	47
t-RNA	10
Unusual DNA	21
Protein-Nucleic Acid Complexes	66
Unusual RNA	14

**Table 2a:** Primary experimental information stored in the NDB

Structure summary^a^	Descriptor
	NDB. PDB, and CSD names
	Coordinates available (yes/no)
	Modifiers (yes/no)
	Mismatches (yes/no)
	Drugs (yes/no)
Structural description^a^	Sequence
	Structure type (A/B/Z/RH/U/P)
	Description of modifiers of base, phosphate, and sugar
	Description of base mismatch
	Name and binding type of drug
	Description of base pairing
	Description of contents of asymmetric unit
Citation^a^	Authors
	Title
	Journal
	Volume
	Pages
	Year
Crystal data^a^	Cell dimensions
	Space group
Data collection description^a^	Source of radiation
	Data collection device
	Radiation wavelength
	Temperature
	Resolution range
	Total and unique number of reflections
Crystallization description^a^	Method
	Temperature
	pH value
	Composition of solutions
Refinement information^a^	Method
	Program
	Number of reflections used for refinement
	Data cutoff
	Resolution range
	R-factor
	Refinement of temperature factors and occupancies
Coordinate information^b^	Atomic coordinates, occupancies and temperature factors forasymmetric unit
	Coordinates for symmetry related strands
	Symmetry related coordinates in unit cell (packing)
	Orthogonal or fractional coordinates

**Table 2b:** Derivative information stored in the NDB

Distances^a^	Chemical bond lengths
	Virtual bonds (involving phosphorus atoms)
Torsions^c^	Backbone and side chain torsion angles
	Pseudorotational parameters
Angles^a^	Valence bond angles
	Virtual angles (involving phosphorus atoms)
Base morphology^a^	Parameters calculated by different algorithms

a Reports can be generated in either ASCII or LATEX.

b Reports can be generated as an NDB or PDB coordinate file, a Kinemage template, or as PostScript molecular graphics.

c Parameters can be displayed in both LATEX or ASCII tables, or as a PostScript conformation wheel.

**Table 3a:** Example 1: Structure selection ofB-DNAs containing the residue sequence “C G C G” without base modifiers, mismatches, or drugs

Table	Property	Operator	Operand	Logical
structural_information	structure_type	–	B	AND
stractural_information	Sequence_of_Strand_A	like	%CGCG%	AND
structurc_summary	base_modifier	is null		AND
structure_summary	mismatch	is null		AND
struaurc_summary	drug	is null		AND

**Table 3b:** Example 2: Structure selection of B-DNAs with resolution ≤1.9 Å and R factors <0.17 by authors A. Rich, R. E. Dickerson, or O. Kctmard

Table	Property	Operator	Operand	Logical
stroctural_information	structure_type	–	B	AND
r_factor	Up_Lim_Resol_Ref	≤	1.9	AND
r_factor	R_Value	<	0.17	AND
citation	authors	like	R. E. Dickerson	OR
slructural_information	structure_type	–	B	AND
r_factor	Up_Lim_Resol_Ref	≤	1.9	AND
r_factor	R_Value	<	0.17	AND
citation	authors	like	A. Rich	OR
stractural_inforrnation	structurejype	-	B	AND
r_factor	Up_Lim_Resol_Ref	≤	1.9	AND
r_factor	R_Value	<	0.17	AND
citation	authors	like	O. Kennard	
